# Abnormalities in the SIRT1-SIRT3 axis promote myocardial ischemia-reperfusion injury through ferroptosis caused by silencing the PINK1/Parkin signaling pathway

**DOI:** 10.1186/s12872-023-03603-2

**Published:** 2023-11-27

**Authors:** Yunfei Liao, Ben Ke, Xiaoyan Long, Jianjun Xu, Yongbing Wu

**Affiliations:** 1 Department of Cardiovascular Surgery, The Second Affiliated Hospital of Nanchang University, Nanchang, China; 2 Department of Nephrology, The Second Affiliated Hospital of Nanchang University, Nanchang, China; 3 East China Digital Medical Engineering Research Institute, Shangrao, China

**Keywords:** SIRT1-SIRT3 axis, Ferroptosis, Mitophagy, PINK1/Parkin signaling pathway, Myocardial ischemia-reperfusion injury

## Abstract

**Background:**

Myocardial ischemia-reperfusion injury (MIRI) is one of the main reasons for poor prognosis in patients with ischemic cardiomyopathy (ICM). To date, the mechanism remains unknown. As members of the silent information regulator 2 (SIR2) family, both SIRT1 and SIRT3 have been shown to play critical roles in protecting cardiomyocytes against MIRI, but their specific protective mechanism, their interact between the two and their relationship with ferroptosis are still unclear. Hence, in this study, we investigated the interact and specific mechanism of SIRT1 and SIRT3 in protecting cardiomyocytes against MIRI, as well as their association with ferroptosis.

**Methods:**

Bioinformatics analysis methods were used to explore the expression of SIRT1 and SIRT3 during MIRI, and then a cell hypoxia/reoxygenation injury model was constructed to verify the results. Then, Pearson correlation analysis was further used to explore the relationship between SIRT1 and SIRT3, whose roles in the regulation of ferroptosis were also analysed by gene knock down, Western Blotting and flow cytometry. Several biomarkers, such as Fe^2+^ concentration, lipid peroxidation marker MDA and mitochondrial membrane potential (MMP), were used to evaluate changes in ferroptosis.

**Results:**

The expression of SIRT1 and SIRT3 was abnormal during MIRI, and SIRT1 was significantly negatively correlated with SIRT3 in the SIRT1-SIRT3 axis. Further analysis revealed that the SIRT1-SIRT3 axis was closely correlated with ferroptosis, and its silencing effectively increase the incidence of ferroptosis. Furthermore, SIRT1-SIRT3 axis silencing was accompanied by changes in PINK1, Parkin, P62/SQSTM1 and LC3 expression. PINK1 silencing significantly increased the incidence of ferroptosis, while resveratrol (Res) and/or honokiol (HKL) effectively reversed the outcome.

**Conclusion:**

Abnormalities in the SIRT1-SIRT3 axis promote MIRI through ferroptosis caused by silencing the PINK1/Parkin signaling pathway.

**Supplementary Information:**

The online version contains supplementary material available at 10.1186/s12872-023-03603-2.

## Background

Ischemic cardiomyopathy (ICM) has become a global problem that threatens human life and health, and its incidence is increasing yearly. More than 90% of ICM is caused by coronary stenosis and blockage [1]. Clinically, thrombolysis, coronary intervention and coronary artery bypass grafting are used to restore ischemic myocardial blood flow (reperfusion), which significantly reduces the mortality of patients. However, studies have shown that reperfusion can lead to myocardial cell energy metabolism disorders, myocardial cell necrosis, apoptosis and structural damage, which further exacerbate myocardial damage, resulting in the expansion of the myocardial necrosis area and even heart failure, and this process is known as myocardial ischemia‒reperfusion injury (MIRI) [2]. Some studies have shown that fatal MIRI can account for up to 50% of the final mortality rate of myocardial infarction [3], MIRI has become an important cause of poor prognosis in patients with ICM, and there are still no effective treatments.

Notably, mitophagy imbalance and subsequent ferroptosis are important mechanisms of MIRI. Substantial evidence suggests that mitochondria are an important target for cardioprotection, and mitochondrial mass impairment is an important factor in MIRI [4]. Mitophagy is central to the mitochondrial quality control system [5]. During MIRI, the mitochondrial mass in cardiomyocytes is damaged, resulting in an imbalance in mitophagy, which further leads to increased ferroptosis [6]. Therefore, how to regulate and maintain the dynamic balance of mitophagy to reduce the incidence of ferroptosis is the key to preventing and treating MIRI.

The Sirtuin protein family are nicotinamide adenine dinucleotide (NAD^+^)-dependent deacetylases [7]. There are 7 members in total: SIRT1 to SIRT7. Their cytoprotective effects are increasingly prominent, and current research has focused on SIRT1 and SIRT3. SIRT1 is mainly located in the nucleus and regulates oxidative stress, cellular metabolism, autophagy and apoptosis through deacetylation [8]. SIRT3 is a mitochondrial protein that regulates mitochondrial protease activity and maintains mitochondrial homeostasis and energy balance [9]. SIRT1 and SIRT3 dually regulate mitochondrial mass, especially of the dynamic balance of mitophagy [8–11]. However, previous studies have mostly focused on either SIRT1 or SIRT3 individually, and few studies have examined the relationship between the two, nor have the relevant mechanisms been examined further. Ilaria Carnevale [12] et al. first proposed the concept of SIRT1-SIRT3 axis. But he did not fully elaborate on the specific interaction between SIRT1 and SIRT3. In the present study, we aimed to investigate the expression and correlation of SIRT1 and SIRT3 in cardiomyocytes during MIRI, and further examined their role in regulating mitophagy balance and ferroptosis, as well as the underlying cardioprotective mechanisms.

## Methods

### Data introduction and preprocessing

“Cardiomyocytes” and “ischemia-reperfusion injury” as keywords were used to retrieve and select datasets that met the requirements. The Gene Expression Omnibus (GEO) database was searched (https://www.ncbi.nlm.nih.gov/geo/). The “GEOquery” package of R software [13] (version 4.0.4, http://r-project.org/) was used to download the GSE5406, GSE1869, GSE974, GSE48060, GSE116250 and GSE97320 datasets. Data from ICM tissues and peripheral blood samples in the form of expression chip data were collected. GSE116250, GSE5406, GSE974 and GSE1869 were obtained from the myocardial tissue of patients with ICM, while GSE48060 and GSE97320 were obtained from the peripheral blood of patients with ICM. The expression profiles were converted and standardized by log_2_ to obtain a series matrix file. The microarray data of GSE5406, GSE1869 and GSE974 were based on the GPL96 platform ([HG-U133A] Affymetrix Human Genome U133A Array) and that of GSE48060 and GSE97320 were based on the GPL570 platform ([HG-U133_Plus_2] Affymetrix Human Genome U133 Plus 2.0 Array). GSE116250 were based on the GPL16791 platform (Illumina HiSeq 2500 (*Homo sapiens*)). High-throughput RNA sequencing (HTS) was performed on all samples. The myocardial tissues of patients with ICM were obtained from biopsies or transplantation; control myocardial tissues were obtained from patients who needed biopsies or transplantation due to other diseases. The peripheral blood samples of experimental group were obtained from patients with ICM, while those in the control group were obtained from patients with other diseases.

### Data merging and principal component analysis (PCA)

The “affy” package [14] was used to read the raw data of the datasets, and the robust multi-array average (RMA) algorithm (www.Bioconductor.org) was used to correct the background and normalize the data [15]. Then, the datasets were merged depending on different classifications using the “merge” function in the “python” package of R software. Among these datasets, GSE5406, GSE1869 and GSE974 from tissue samples belonged to one category, and the other category included GSE48060 and GSE97320 from peripheral blood samples. After merging the data, the “sva” package in R software was used to remove batch effects on the merged datasets. PCA is a multiple regression analysis and was used to assess the merged data [16]. The “factoextra” package in R software was used for data processing, analysis and mapping. The effect of data correction was demonstrated using a two- or three-dimensional PCA cluster plot.

### SIRT1/SIRT3 expression and Pearson correlation analysis

After merging the data and removing batch effects, the microarray data of SIRT1 and SIRT3 were extracted into a separate matrix file. The expression of SIRT1 and SIRT3 in the ICM and control groups were analysed, and the results were visualized with ‘ggboxplot’ package. Then a Pearson correlation analysis for the expression of SIRT1 and SIRT3 in the ICM and control groups was performed. The R package ‘ggscatter’ was used to visualize the correlation of SIRT1 and SIRT3 expression in the two groups.

### Cell culture

The immortalized rat cardiomyocyte cell line H9c2 was purchased from Wuhan Procell Life Technology Co., Ltd (CL-0089). H9c2 cells were cultured in Dulbecco’s modified Eagle medium (DMEM) supplemented with 10% fetal bovine serum (FBS) and a 1% penicillin‒streptomycin solution. The cells were cultured in an incubator at a constant temperature of 37 °C and 5% carbon dioxide. The cells were passaged when they reached 70–80% confluence. Third to fifth passage H9c2 cells were used for the experiments. H9c2 cells after treatment were digested with 0.25% trypsin and harvested.

### Construction of a hypoxia/reoxygenation (H/R) injury cell model in vitro

H9c2 cells were subjected to 3 h of hypoxia followed by 16 h of reoxygenation at 37 °C. To simulate hypoxia, the cell culture medium was replaced with Tyrode solution containing the following (in mmol/L): 130 NaCl, 5 KCl, 10 HEPES, 1 MgCl_2_, 1.8 CaCl_2_ at pH 7.4/37°C. The cells were exposed to hypoxia in a controlled hypoxic chamber (0x-101 C-HP, Shanghai Tawang Intelligent Technology Co., Ltd; Shanghai, China) with a 1% O_2_, 94% N_2_ and 5% CO_2_ gas mixture flushing up to a partial O_2_ pressure of 1%. Reoxygenation was conducted in a normoxic incubator at 37 °C for 16 h, and the ischemia medium was replaced with DMEM supplemented with 10% FBS. The control group consisted of cells that were not exposed to hypoxia and were kept in the reoxygenation buffer.

### Flow cytometric analysis of apoptosis

H9c2 cells were harvested 24 h after H/R treatment. The cells were washed twice with ice-cold phosphate buffer solution (PBS) and resuspended in binding buffer. The cell suspension was transferred into a tube and double-stained with Annexin V-FITC and propidium iodide (PI) at room temperature in the dark for 15 min, according to the instructions of the Annexin V apoptosis detection kit (US Everbright, San Ramon, CA, USA). The percentage of apoptotic cells was quantified by flow cytometry at 530 and 600 nm to measure green Annexin V-FITC and red PI fluorescence respectively (FACSCalibur, BD Biosciences, San Jose, CA, USA). Further differentiation between early and late apoptosis was determined by Annexin V and PI staining in different quadrants: AV+/PI + indicated late-phase apoptotic cells, and AV+/PI- indicated early-phase apoptotic cells.

### Mitochondrial membrane potential (MMP) assay with JC-1

JC-1 is an ideal fluorescent probe that is widely used to detect MMP. The cell collection and pretreatment steps were the same as those used for flow cytometric analysis. The cell suspension was transferred into a tube and stained with JC-1 at room temperature in the dark for 15 min, according to the instructions of the JC-1 MMP detection kit (Cat No: KGA604, KeyGen BioTECH, Nanjing, China). The percentage of cells with damaged mitochondria was quantified by flow cytometry at 530 and 600 nm to measure green FITC and red PE fluorescence, respectively (FACSCalibur, BD Biosciences, San Jose, CA, USA). Further differentiation between normal and damaged cells was determined by JC-1 staining in different quadrants: FITC+/PE + indicated cells with normal mitochondria, and FITC+/PE- indicated cells with damaged mitochondria.

### Western blotting

H9c2 cell proteins were extracted with lysis buffer. Total protein concentrations were then quantified with a BCA protein assay kit (Cwbiotich, Peking, China). Equivalent amounts of protein (30 µg per lane) were loaded and separated by 10% SDS‒PAGE gels and transferred to polyvinylidene difluoride (PVDF) membranes. The membranes were blocked with 5% skimmed milk solution in Tris-buffered saline with 0.1% Triton X-100 (TBST) for 1 h at room temperature and then incubated overnight at 4 °C with primary antibodies against SIRT1 (1:1000, ab189494, Abcam, Cambridge, MA, USA), SIRT3 (1:1000, D22A3, CST, Danvers, Massachusetts, USA), PINK1 (1:500, GTX107851, GeneTex, Southern California, USA), Parkin (1:500, Cat No. 381,626, Zenbio, Peking, China), LC3-A/B (1:1000, Cat No. 206,019, Zenbio, Peking, China), P62/SQSTM1 (1:1000, ab109012, Abcam, Cambridge, MA, USA) and GAPDH (1:10000, ab181602, Abcam, Cambridge, MA, USA). After the membranes were washed three times, they were incubated with secondary antibodies (1:10000, Lot 01334 and Lot 01325, Cwbiotech, Nanjing, China) for 1 h at room temperature. Immunoreactivity was detected with Super Signal West Pico Chemiluminescent Substrate (GelView 6000 M, Guangzhou Yunxing Scientific Instrument Co., Ltd, Guangzhou, China) and the band intensities were evaluated with ImageJ. The images of the blots were simply cropped, and the original images of the uncropped blots are displayed in the supplementary information (Supplementary Original blot images).

### Transfection and gene knockdown

Specific small hairpin RNAs (shRNAs) targeting SIRT1 (shSIRT1) and SIRT3 (shSIRT3) and small interfering RNAs (siRNAs) targeting PINK1 (siPINK1), which should not knock down any known proteins, were purchased from Jiangsu KeyGEN BioTECH Co., Ltd (Nanjing, China). These shRNA or siRNA molecules were transfected with Lipofectamine TM3000 (Invitrogen, CA, USA) for 48 h. After transfection and knockdown verification, the sequences with the best knockdown efficiency were selected for subsequent experiments. After the target genes were knocked down for 24 h, the cells were subjected to H/R and used for various measurements.

### Differentially expressed genes (DEGs) screening

Data collection and preprocessing were the same as described above. 218 ferroptosis-related genes were extracted from the GSE116250 dataset using R software. The effect of data correction was shown using a two-dimensional PCA cluster plot. DEGs were screened by the “limma” package [17], and heatmap and volcano maps of DEGs were drawn using the “ggplot2” package to visualize the differential expression levels. DEGs with *p* < 0.05 and |log_2_ FC| > 0.8 were considered statistically significant.

### Least absolute shrinkage and selection operator (LASSO) logistic regression and Pearson correlation analysis

LASSO logistic regression [18] was used to perform feature selection to screen the core genes related to ferroptosis. The LASSO algorithm was applied by the “glmnet” package [19]. Then, Pearson correlation analysis of the core genes and SIRT1/SIRT3 was further performed by the “corrplot” package, and the “ggplot2” package was used to draw a chord diagram to visualize the results. A two-sided *p* < 0.05 was considered statistically significant.

### Determination of Fe^2+^ concentrations

After gene knockdown and H/R treatment, H9c2 cells were harvested as described above. Equal amounts of cells were taken from each group, washed twice with ice-cold PBS, resuspended in lysis buffer, and lysed on ice for 2 h. Then, the cells were centrifuged at 13,000 rpm for 10 min at 4 °C, and the supernatant was collected. The Fe^2+^ concentrations were determined according to the instructions of the Fe^2+^ concentration assay kit (E1042, Applygen, Peking, China).

### Malondialdehyde (MDA) detection

After gene knockdown and H/R treatment, H9c2 cells were harvested as described above. After washed twice with ice-cold PBS, the cells were resuspended in lysis buffer, and lysed on ice for 2 h. Then, the protein concentrations of each group were determined according to the instructions of the BCA protein concentration assay kit (CW0014S, Cwbiotech, Beijing, China). MDA concentrations in each group were determined according to the instructions of the MDA concentration determination kit (Beyotime Biotechnology, Shanghai, China). The ratio between the measured MDA concentration in each group and the protein concentration of the corresponding group was the standardized MDA value (nmol/mg).

### Autophagy-related genes datasets and Pearson correlation analysis

A total of 215 autophagy-related genes were obtained from The Human Autophagy Database (http://www.autophagy.lu/index.html). The microarray data of autophagy-related genes were extracted from the GEO116250 dataset. Pearson correlation analysis was performed for SIRT1/SIRT3 and the 215 autophagy-related genes. A two-sided *p* < 0.05 was considered statistically significant.

### Statistical analysis

All experiments were repeated independently at least 3 times. Most of the statistical analysis was performed using GraphPad Prism software (version 9.0.0, GraphPad, La Jolla, CA, USA), while statistical analysis of the bioinformatics data was conducted by R software. The Shapiro–Wilk test was used to test the normality of the data. All normally distributed data are presented as the means ± SD. For comparisons between multiple groups, quantitative normal variables were analysed by one-way ANOVA followed by Tukey’s test for multiple comparisons, and nonnormal variables were analysed by the Kruskal–Wallis test followed by Dunn’s test. Comparisons between two groups were performed by unpaired Student’s t tests. A two-sided value of *p* < 0.05 was considered statistically significant. Pearson’s correlation test was used to analyse the correlation.

## Results

### Abnormal expression of SIRT1 and SIRT3, and mutual inhibition between the two

**Fig. 1:**
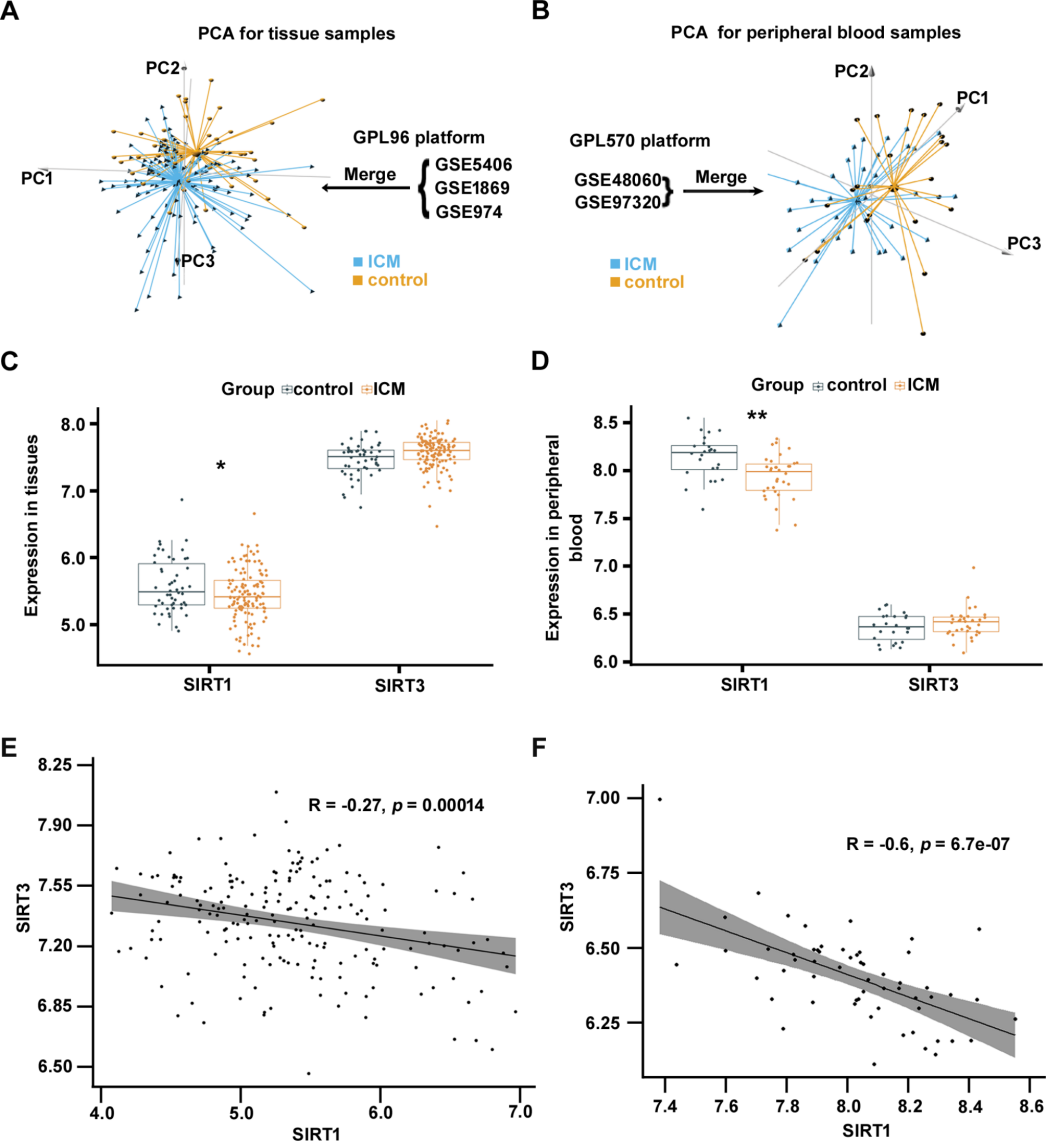
Expression and Pearson correlation analysis of SIRT1 and SIRT3 in tissues and peripheral blood. (**A, B**) PCA of merged datasets obtained from tissue and peripheral blood samples of patients with ICM. (**C, D**) Expression of SIRT1 and SIRT3 in tissues and peripheral blood. (**E, F**) Pearson correlation analysis of SIRT1 and SIRT3 in tissues and peripheral blood. *p < 0.05, **p < 0.01

After preprocessing the datasets, the homogeneity of the merged datasets before and after the removal of batch effects was examined, and the results showed that the batch effects were removed completely (Supplemental Fig. S1A-D). Then, three-dimensional PCA was performed on the merged datasets, and the results showed that the samples were subclustered well (Fig. 1A, B). Subsequently, the expression of SIRT1 and SIRT3 in myocardial tissues and the peripheral blood of patients with ICM was analysed, as well as their expression correlations. The results showed that the expression of SIRT1 in myocardial tissues and peripheral blood in the ICM group was significantly lower than that in the control group (*p* < 0.05), and no significant difference was observed in SIRT3 (*p* > 0.05) (Fig. 1C, D). Extremely significant negative correlations between SIRT1 and SIRT3 were observed in myocardial tissues and peripheral blood samples (Fig. 1E, F) (*p* < 0.001).

**Fig. 2:**
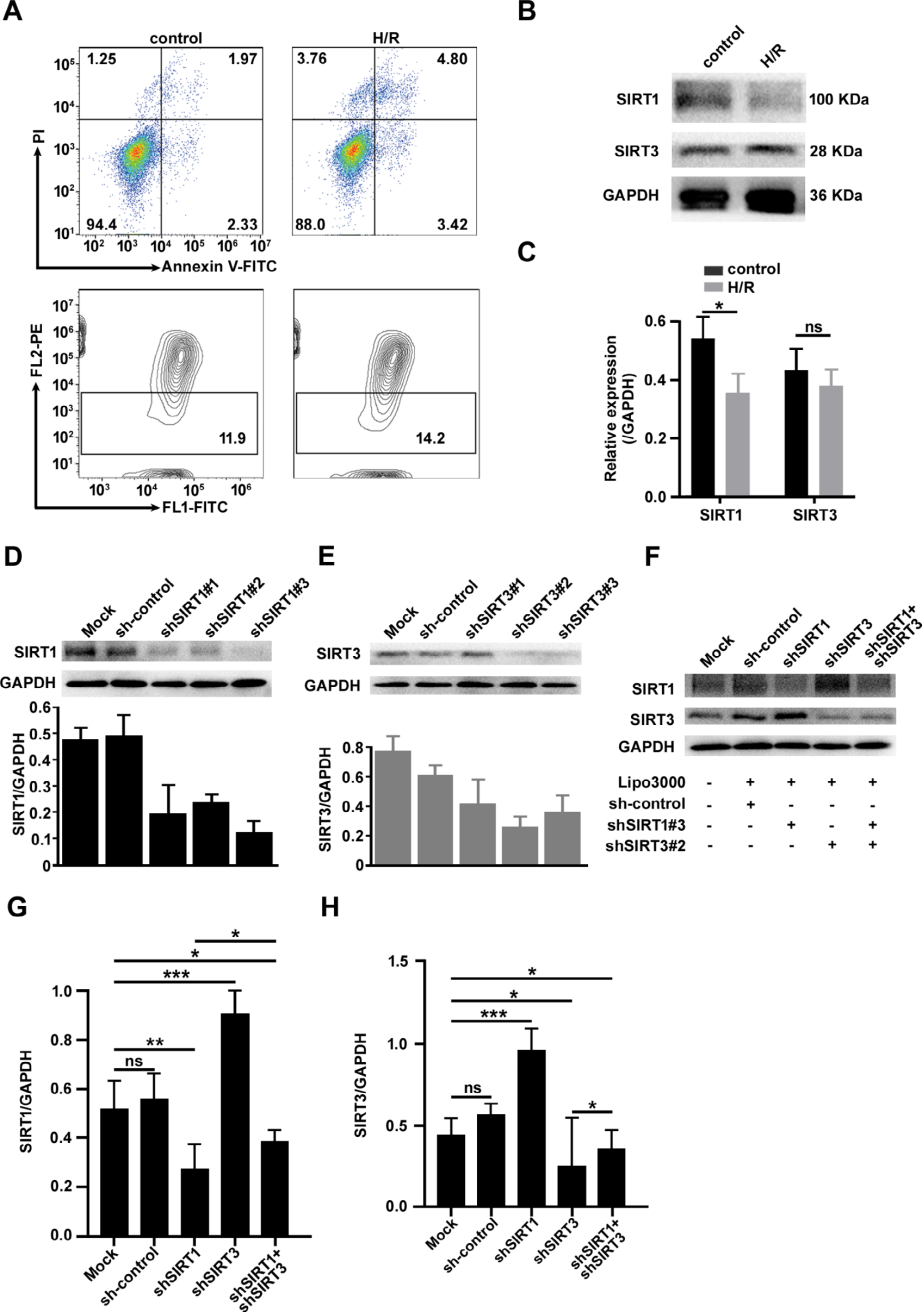
Validation of SIRT1 and SIRT3 expression and their correlation in an in vitro H/R injury cell model. (**A**) Flow cytometric analysis of apoptosis and MMP detection in the H/R and control groups. (**B, C**) The protein levels of SIRT1 and SIRT3 were measured by Western blotting in the H/R and control groups. (**D, E**) The protein levels of SIRT1 and SIRT3 were measured by Western blotting after shRNA infection. (**F-H**) Representative images of Western blot analysis of SIRT1 and SIRT3 expression after shRNA infection and quantitative results. The data are presented as the mean ± SD, n = 3–5. *p < 0.05, **p < 0.01, ***p < 0.001, and ns: *p* > 0.05. Lipo3000: Lipofectamine TM3000, a reagent for auxiliary transfection

To clarify the expression of SIRT1 and SIRT3 and their correlation in cardiomyocytes during MIRI, we constructed an in vitro H/R cell model to simulate MIRI. After the cell model was constructed, apoptosis and MMP were used to examine and verify the establishment of this cell model, and the results demonstrated that the H/R cell model was successfully induced in vitro (Fig. 2A). Then, the cell model was used to examine the protein expression of SIRT1 and SIRT3, and the results were consistent with the gene expression (Fig. 2B, C). The results showed that the expression of SIRT1 in the H/R group was lower than that in the control group (*p* < 0.05), and no significant difference was observed in the expression of SIRT3 in the two groups (*p* > 0.05). Subsequently, the expression of SIRT1 and SIRT3 were knocked down in the H/R cell model using shRNAs. Three shRNAs, were designed and the sequences are shown in Table 1. After fully optimizing the infection conditions, it was found that shSIRT1#3 and shSIRT3#2 were the sequences with the best effects on gene knockdown among the three shRNA sequences targeting SIRT1 and SIRT3 (Fig. 2D, E). Therefore, shSIRT1#3 and shSIRT3#2 were selected for subsequent experiments.

**Table 1:** shRNA sequences targeting SIRT1 and SIRT3 used in this study

Names		Sequences (5’→3’)
shSIRT1#1	Sense	CCAGUAGCACUAAUUCCAATT
	Antisense	UUGGAAUUAGUGCUACUGGTC
shSIRT1#2	Sense	CCCUGUAAAGCUUUCAGAATT
	Antisense	UUCUGAAAGCUUUACAGGGTT
shSIRT1#3	Sense	GCUACACUUGUAGACCAAATT
	Antisense	UUUGGUCUACAAGUGUAGCAA
shSIRT3#1	Sense	CCAUCUUUGAACUAGGCUUTT
	Antisense	AAGCCUAGUUCAAAGAUGGTT
shSIRT3#2	Sense	UACCCUGAGGCCAUCUUUGAATT
	Antisense	UUCAAAGAUGGCCUCAGGGUATT
shSIRT3#3	Sense	CUUGUCUGAAUCGGUACAGAATT
	Antisense	UUCUGUACCGAUUCAGACAAGTT

**Note**: shRNA, short hairpin RNA

Five groups were created to assess the relationship between the protein expression of SIRT1 and SIRT3. The five groups were the blank control group (Mock), sham treatment group (sh-control), shSIRT1 group (gene knockdown of SIRT1), shSIRT3 group (gene knockdown of SIRT3) and shSIRT1 + shSIRT3 group (gene knockdown of both SIRT1 and SIRT3). The expression levels of SIRT1 and SIRT3 in each group were determined by Western blotting (Fig. 2F). Interestingly, it was observed that the expression of SIRT1 in the shSIRT3 group increased significantly (*p* < 0.001) (Fig. 2G), and the expression of SIRT1 in the shSIRT1 + shSIRT3 group was also higher than that in shSIRT1 group (*p* < 0.05). Surprisingly, the expression of SIRT3 in the shSIRT1 group was significantly increased (*p* < 0.001) (Fig. 2H), and the expression of SIRT3 in the shSIRT1 + shSIRT3 group was higher than that in the shSIRT3 group (*p* < 0.05). These results suggest that the expression levels of SIRT1 and SIRT3 are abnormal, and there is a negative correlation between SIRT1 and SIRT3, which verifies the existence of the SIRT1-SIRT3 axis.

### Abnormalities in the SIRT1-SIRT3 axis cause MIRI through ferroptosis

**Fig. 3:**
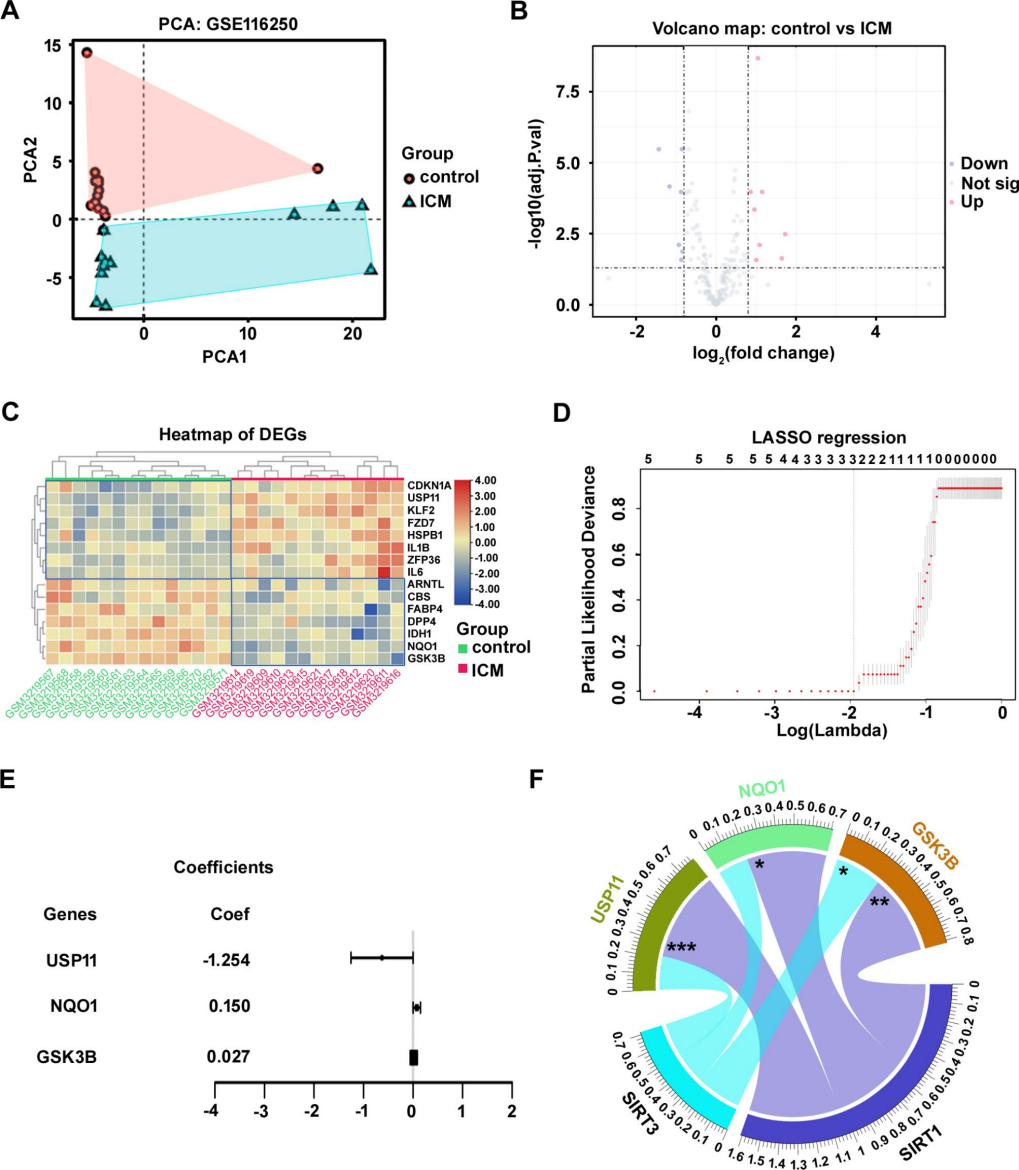
The SIRT1-SIRT3 axis is closely correlated with ferroptosis. (**A**) PCA of dataset GSE116250. (**B, C**) Volcanic map and clustering heatmap of DEGs related to ferroptosis. (**D, E**) LASSO regression model analysis. The dotted line shows the best lambda. USP11, NQO1 and GSK3B are core DEGs screened by LASSO. (**F**) Chord diagram of Pearson correlation analysis results of SIRT1/SIRT3 and the 3 core DEGs. *p < 0.05, **p < 0.01, ***p < 0.001

Previous studies have demonstrated that the protective effect of the Sirtuin family on cardiomyocytes involves the modulation of ferroptosis, but the specific mechanism is still unclear [20, 21]. In this study, the relationship between the SIRT1-SIRT3 axis and ferroptosis in cardiomyocytes during MIRI was examined. The GSE116250 dataset from the GEO database was selected. After standardization, the expression matrix was uploaded for PCA, and the results showed that the data were subclustered well (Fig. 3A). Then, the microarray data of 218 ferroptosis-related genes [22] from the GSE116250 dataset was extracted (Supplemental Table S1). Subsequently, the DEGs related to ferroptosis between the ICM and control groups were extracted, and the results were visualized by a volcano map and clustering heatmap (Supplemental Table S2; Fig. 3B, C). There were 15 DEGs, including 8 upregulated genes and 7 downregulated genes. Then, 3 core DEGs were further screened with LASSO algorithm from the 15 DEGs (Fig. 3D, E). The 3 core DEGs were USP11, NQO1 and GSK3B. Finally, a Pearson correlation analysis of SIRT1/SIRT3 and the 3 core DEGs was performed. The chord diagram of the Pearson correlation analysis results showed that SIRT1/SIRT3 was closely correlated with the 3 core DEGs (Fig. 3F), which indicates that the SIRT1-SIRT3 axis participates in modulating ferroptosis in cardiomyocytes during MIRI.

**Fig. 4:**
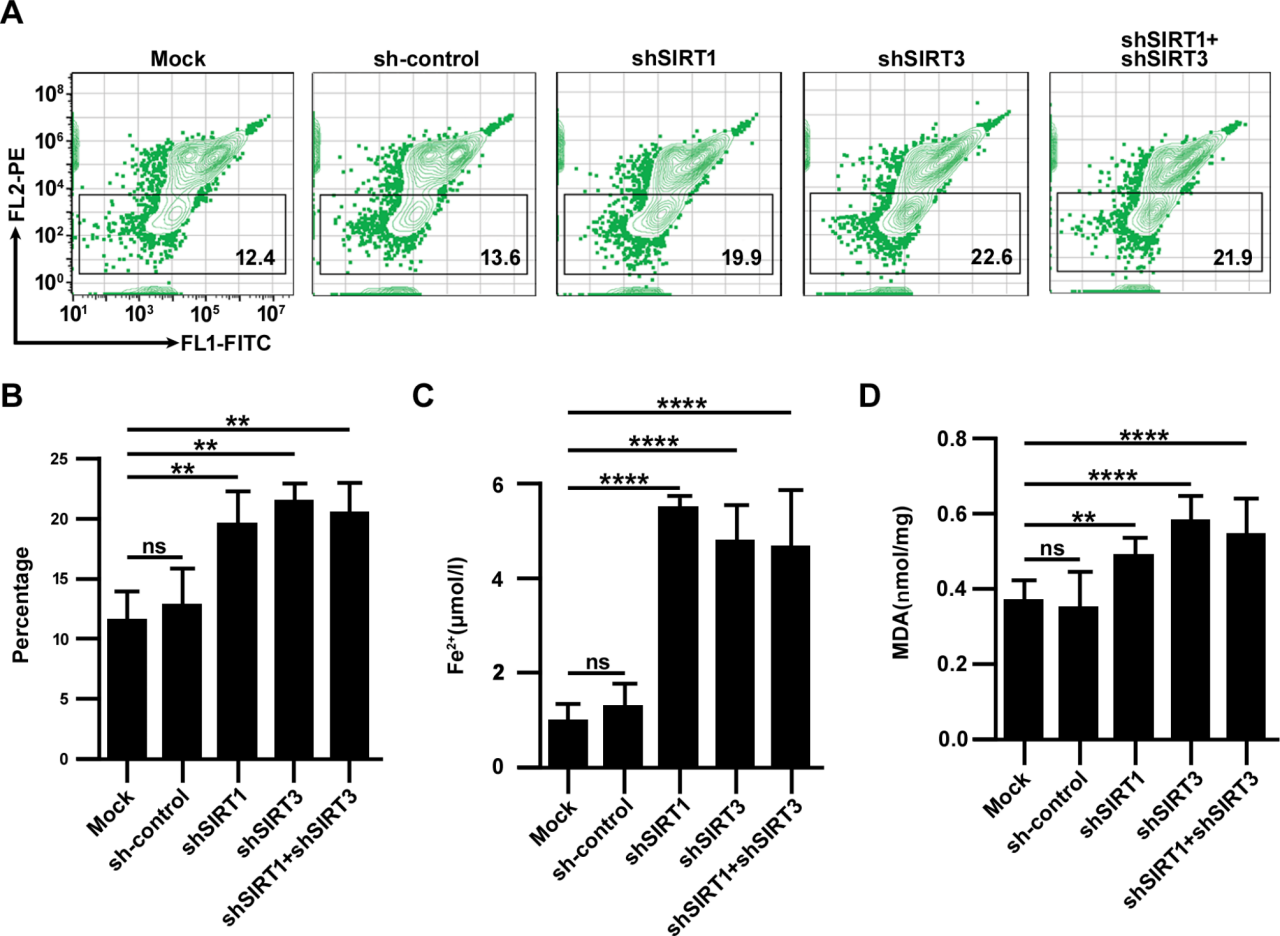
The incidence of ferroptosis increased after SIRT1-SIRT3 axis silencing. (**A, B**) The MMP was detected by JC-1 in each group after shRNA infection and H/R, which represents the proportion of damaged mitochondria. (**C**) Fe^2+^ concentrations. (**D**) MDA contents. The data are presented as the mean ± SD, n = 3–5. *p < 0.05, **p < 0.01, ***p < 0.001, ****p < 0.0001, and ns: *p* > 0.05

Subsequently, the specific role of the SIRT1-SIRT3 axis in cardiomyocyte ferroptosis during H/R injury was further examined. Ferroptosis is characterized by Fe^2+^ accumulation and lipid peroxidation, which are accompanied by changes in MMP. Therefore, several biomarkers, such as Fe^2+^ concentration, lipid peroxidation marker MDA and MMP, were selected to evaluate changes in ferroptosis. It was demonstrated that gene knockdown of SIRT1 and/or SIRT3 increased mitochondrial damage (Fig. 4A, B) (*p* < 0.01), Fe^2+^ deposition (Fig. 4C) (*p* < 0.0001) and the accumulation of toxic substances such as MDA (Fig. 4D) (*p* < 0.0001), which indicated that SIRT1-SIRT3 axis silencing could reduce the resistance of cardiomyocytes to H/R injury, increasing the incidence of ferroptosis, which suggests that abnormalities in SIRT1-SIRT3 axis lead to MIRI through ferroptosis-dependent cell death. In addition, it was also found from the above results that knockdown either SIRT1 or SIRT3 expression would lead to an increase in the incidence of ferroptosis, which indicates that although SIRT1 and SIRT3 inhibit each other, either of them is indispensable, further confirming the overall attribute of the SIRT1-SIRT3 axis.

### Abnormalities in SIRT1-SIRT3 axis promote MIRI through ferroptosis caused by silencing the PINK1/Parkin signaling pathway

**Fig. 5:**
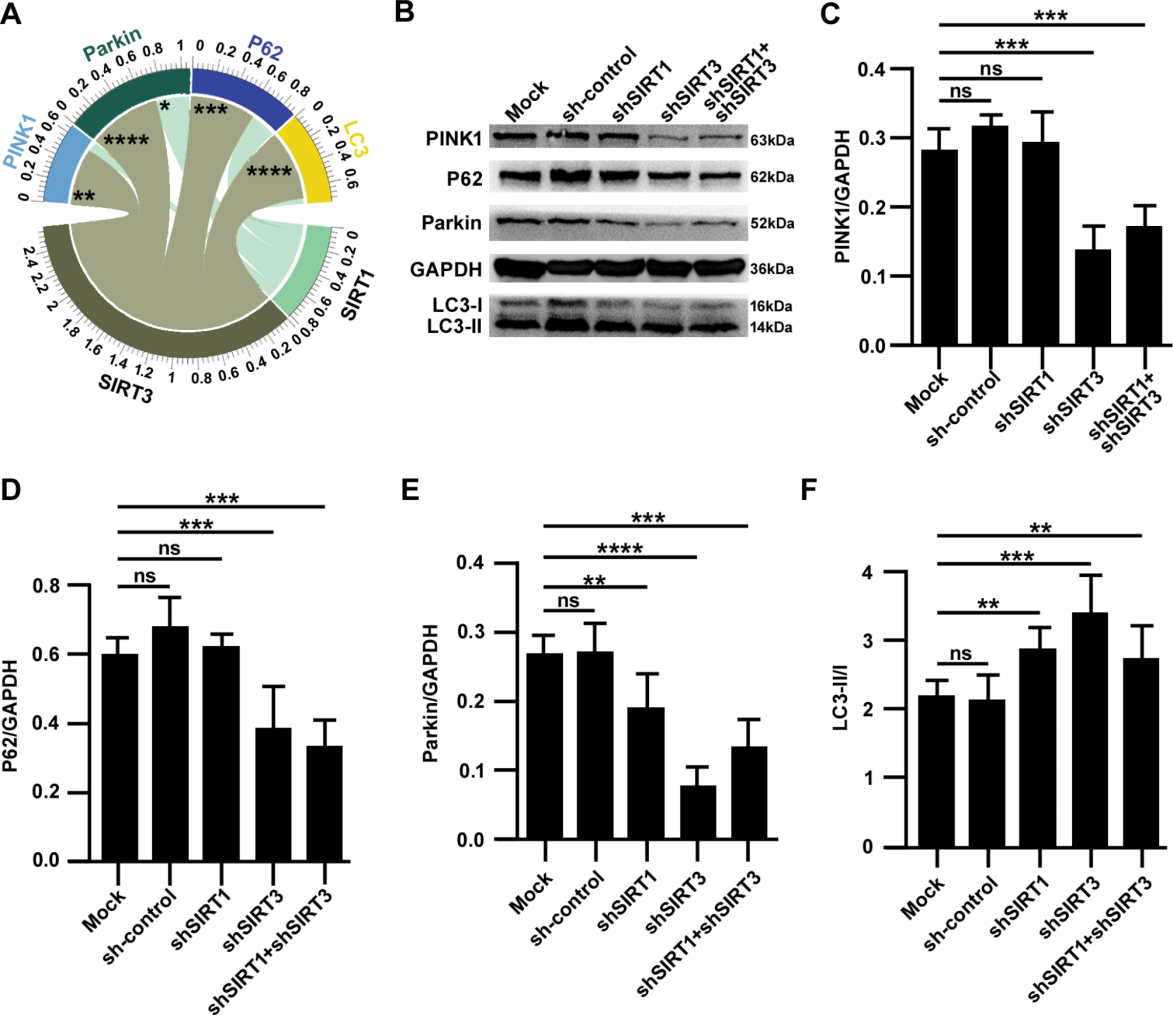
SIRT1-SIRT3 axis silencing is accompanied by changes in PINK1, Parkin, P62/SQSTM1 and LC3 expression. (**A**) The Pearson correlation analysis chord diagram of the SIRT1-SIRT3 axis and PINK1, Parkin, P62/SQSTM1 and LC3. (**B-F**) Representative images of Western blotting and the quantitative results. The data are presented as the mean ± SD, n = 3–5. *p < 0.05, **p < 0.01, ***p < 0.001, ****p < 0.0001, and ns: *p* > 0.05

A disruption in autophagy balance can increase the incidence of ferroptosis [6]. Bioinformatics analysis showed that changes in the expression of SIRT1 and SIRT3 were accompanied by changes in the expression of autophagy-related genes, which suggested that the SIRT1-SIRT3 axis could modulate the incidence of ferroptosis by regulating autophagy balance during MIRI. Thus, the microarray data of 215 autophagy-related genes (Supplemental Table S3) from the GSE116250 dataset was extracted. Pearson correlation analysis showed that SIRT1/SIRT3 was closely correlated with PINK1, Parkin, P62/SQSTM1 and LC3 (Fig. 5A) (*p* < 0.05). To further validate the correlation between SIRT1/SIRT3 and these four genes, Western blotting was performed to detect their expression after SIRT1-SIRT3 axis silencing. It was observed that the expression of these proteins changed after SIRT1-SIRT3 axis silencing (Fig. 5B), which was consistent with the changes in gene expression. The housekeeping gene GAPDH was used as the reference, and it was found that the expression of PINK1 decreased only in groups with SIRT3 gene knockdown (*p* < 0.001), and no significant changes in the other groups were observed (Fig. 5C). The change in P62 expression was consistent with that of PINK1 (Fig. 5D) (*P* < 0.001). The expression of Parkin decreased in all groups with SIRT1 and/or SIRT3 gene knockdown (Fig. 5E) (*p* < 0.01). The LC3-II/I ratio was increased in all groups with SIRT1 and/or SIRT3 gene knockdown (*p* < 0.01) (Fig. 5F). Changes in the expression of these autophagy-related proteins can reflect changes in the autophagy balance [23, 24]. Therefore, these results indicated that the autophagy balance was disrupted due to abnormalities in the SIRT1-SIRT3 axis during MIRI. In other words, abnormalities in the SIRT1-SIRT3 axis cause MIRI by disrupting the autophagy balance.

**Fig. 6:**
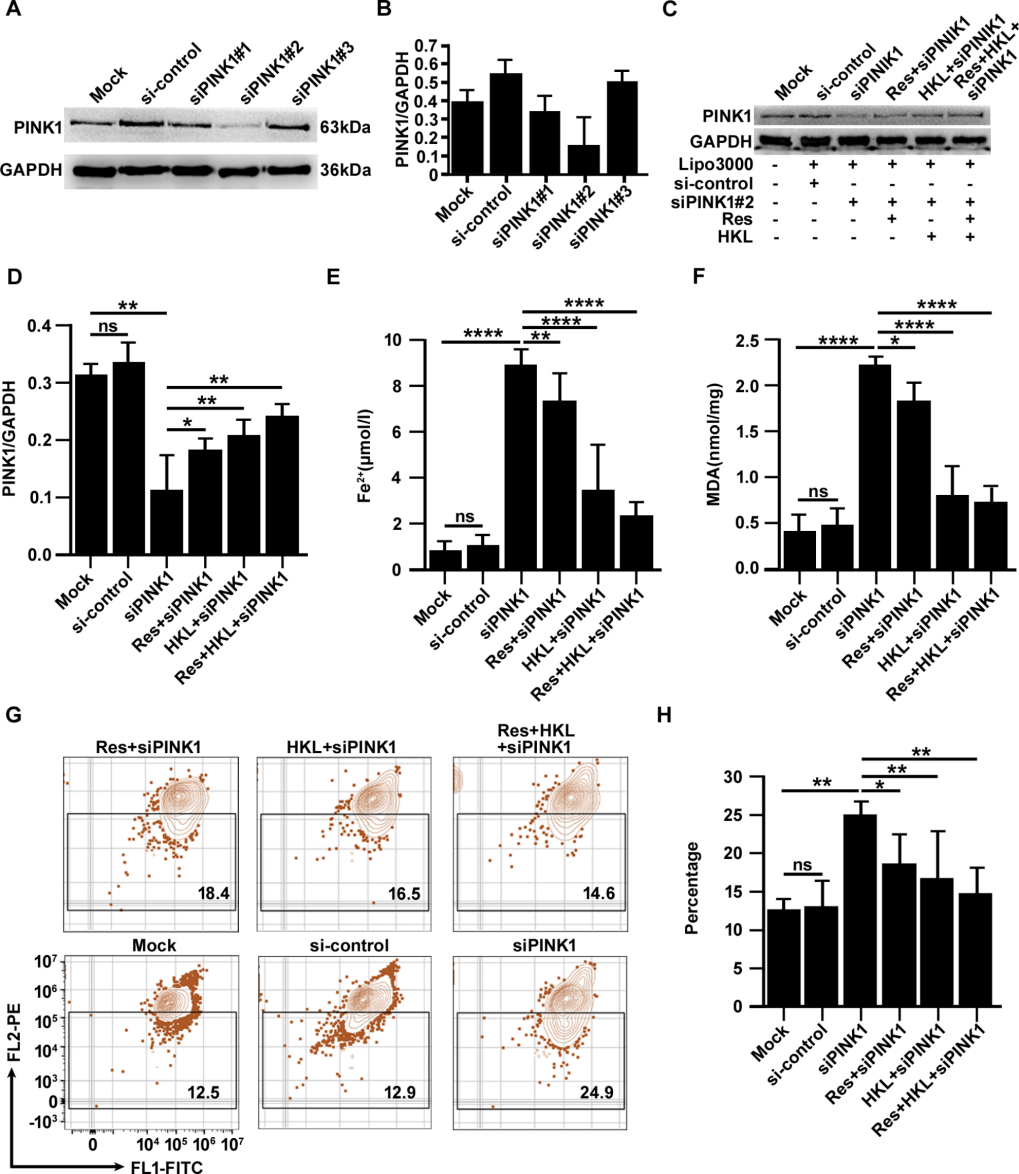
The SIRT1-SIRT3 axis modulates the incidence of ferroptosis via the PINK1/Parkin signaling pathway. (**A, B**) The protein level of PINK1 was measured by Western blotting after siPINK1 infection. (**C, D**) PINK1 expression and quantitative results after siPINK1 infection and treatment. (**E**-**H**) Fe^2+^ concentrations, MDA levels and MMP were measured after siPINK1 infection and treatment. The data are presented as the mean ± SD, n = 3–5. *p < 0.05, **p < 0.01, ***p < 0.001, ****p < 0.0001, and ns: *p* > 0.05. Lipo3000: Lipofectamine TM3000, a reagent for auxiliary transfection. Res: resveratrol. HKL: honokiol

According to these results, we hypothesized that abnormalities in the SIRT1-SIRT3 axis led to MIRI through ferroptosis caused by mitophagy imbalance. To further confirm that the SIRT1-SIRT3 axis modulates the occurrence of ferroptosis by regulating the autophagy balance, the expression of PINK1, the key molecule in the PINK1/Parkin signaling pathway, was knocked down. After gene knockdown, H9c2 cells were treated with resveratrol (Res) and/or honokiol (HKL). The concentration and duration of administration of Res and HKL were 20 nmol/l for 24 h. After H/R injury, H9c2 cells were collected, and ferroptosis-related phenotypes were observed. Six groups were designed: blank control group (Mock), sham treatment group (si-control), siPINK1 (gene knockdown targeting PINK1) group, Res + siPINK1 group, HKL + siPINK1 group, and Res + HKL + siPINK1 group. The small interfering sequences targeting PINK1 are shown in Table 2. After optimized the infection and gene knockdown validation, the sequence with the best knockdown effect was selected for subsequent experiments (Fig. 6A, B).

**Table 2:** siRNA sequences targeting PINK1 used in this study

Names		Sequence (5’→3’)
siPINK1#1	Sense	GCAAUUUUUACACAGAAAATT
	Anti-sense	UUUUCUGUGUAAAAAUUGCTT
siPINK1#2	Sense	GUAAACUGUACAGGAAAUUTT
	Anti-sense	AAUUUCCUGUACAGUUUACTT
siPINK1#3	Sense	CUAUACUCUUCUCAUUUUUTT
	Anti-sense	AAAAAUGAGAAGAGUAUAGTT

**Note**: siRNA, short interfering RNA

After PINK1 gene silencing and treatment, Western blotting was used to determine the protein level of PINK1. The results showed that the expression of PINK1 was significantly decreased (*p* < 0.01), while Res and HKL significantly increased the expression of PINK1 (*p* < 0.05) (Fig. 6C, D). Then, the Fe^2+^ concentration, MDA levels and MMP in each group were measured. After gene silencing and treatment, H9c2 cells exhibited the same ferroptosis-related phenotypes as the SIRT1-SIRT3 axis silencing, such as Fe^2+^ deposition (*p* < 0.0001) (Fig. 6E), the accumulation of the toxic substance MDA (*p* < 0.0001) (Fig. 6F), and increased mitochondrial damage (*p* < 0.01) (Fig. 6G, H). Interestingly, both Res and HKL effectively reversed these effects caused by PINK1 gene silencing, especially HKL (*p* < 0.05). These results suggest that the SIRT1-SIRT3 axis modulates ferroptosis by regulating mitophagy balance via the PINK1/Parkin signaling pathway, confirming the relationship among the SIRT1-SIRT3 axis, ferroptosis and the PINK1/Parkin signaling pathway. Therefore, abnormalities in the SIRT1-SIRT3 axis contribute to MIRI through mitophagy-related ferroptosis caused by silencing the PINK1/Parkin signaling pathway.

## Discussion

**Fig. 7:**
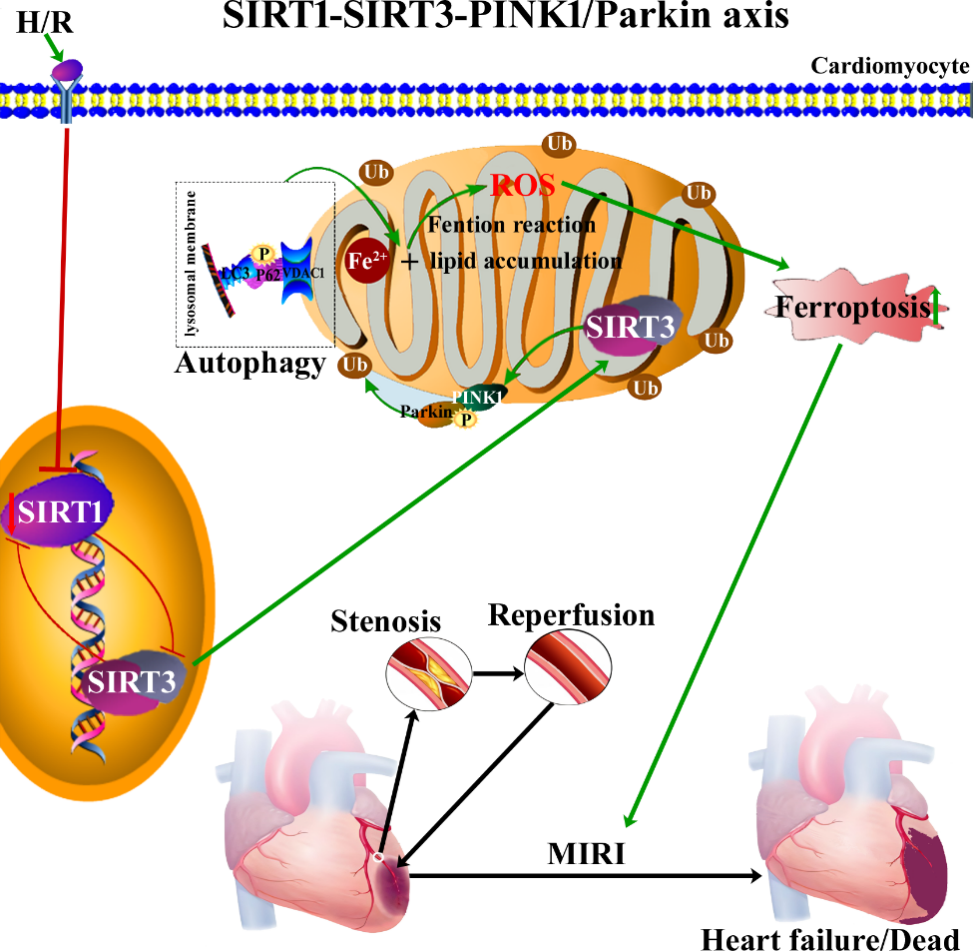
Abnormalities in the SIRT1-SIRT3 axis promote MIRI through ferroptosis caused by silencing of the PINK1/Parkin signaling pathway. The red lines represent an inhibitory effect, while the green lines represent the initiation or entry of the downstream stage. The red downward arrows represent decreases, while the green upward arrows represent increases. ROS: reactive oxygen species, H/R: hypoxia/reoxygenation. Ub: ubiquitination. P: phosphorylation

MIRI is still an intractable clinical problem that needs to be further addressed [25]. MIRI has become an important cause of poor prognosis in patients with ICM. To date, the mechanism of MIRI remains unknown, and there are no effective treatments. Therefore, it is important to elucidate its pathogenesis and identify potential therapeutic targets. In this study, we confirmed that abnormalities in the SIRT1-SIRT3 axis contributed to MIRI through ferroptosis caused by silencing the PINK1/Parkin signaling pathway. Briefly, the expression of the SIRT1-SIRT3 axis is abnormal during MIRI, which consequently causes a silence in the PINK1/Parkin signaling pathway. As a result, mitochondrial ubiquitination was impaired and the mitophagy balance was disrupted in cardiomyocytes, which ultimately led to Fe^2+^ deposition, lipid peroxidation, and increased ROS via the Fenton reaction, which increased the incidence of ferroptosis [26, 27] (summarized in Fig. 7).

As key members of the silent information regulator 2 (SIR2) protein family, both SIRT1 and SIRT3 have been shown to play critical roles in protecting cardiomyocytes against MIRI [28–30]. However, there have been few reports on the specific expression of SIRT1 and SIRT3 when cardiomyocytes suffer from MIRI. In addition, previous studies have mostly focused on either SIRT1or SIRT3, and no research has examined the potential relationship between the two, nor have the relevant mechanisms been examined in depth. The present study investigated the expression and correlation of SIRT1 and SIRT3 in cardiomyocytes suffering from MIRI at gene and protein levels. It was found that the expression of SIRT1 in the tissue and peripheral blood of patients with ICM was lower than that in the control group, while the expression of SIRT3 showed no significant changes. However, further experiments confirmed that SIRT1 and SIRT3 exhibited significant mutual inhibition. Consequently, we hypothesized that it was a false phenomenon that there was no significant change in the expression of SIRT3 when cardiomyocytes suffered from MIRI. In fact, the expression of SIRT3 changed in two opposite ways. One was upregulation due to loss of SIRT1 inhibition, and the other was downregulation due to MIRI, which ultimately led to the expression of SIRT3 showing no significant changes. This point was demonstrated by the significant upregulation of SIRT3 expression after SIRT1 was knocked down. Tseng et al. [31] found that transient oxidative stress could upregulate SIRT3 expression, while sustained stimulation could downregulate SIRT3 expression. Therefore, we concluded that the expression of SIRT1 and SIRT3 was abnormal when cardiomyocytes suffered from MIRI. SIRT1 expression was downregulated, while SIRT3 expression was dynamically altered due to various factors.

Ilaria Carnevale [12] et al. first proposed the concept of SIRT1-SIRT3 axis. However, he did not fully elaborate on the specific interaction between SIRT1 and SIRT3. According to the biological effects of SIRT1 and SIRT3, we speculate that this axis can be used by cells to respond to stress, such as oxidative stress and DNA damage [12, 32]. Previous studies have shown that nuclear SIRT1 can control the expression of SIRT3 through deacetylation and regulating transcription factors that bind to the SIRT3 promoter [12]. Silencing SIRT1 in different cell lines is accompanied by an increase in SIRT3 expression [12]. In this study, we also found that SIRT1 silencing was accompanied by a significant increase in SIRT3 expression. Interestingly, we showed for the first time that silencing SIRT3 was also accompanied by a significant increase in SIRT1 expression, which means that the interaction between SIRT1 and SIRT3 is complex. The specific interaction between SIRT1 and SIRT3 may be a self-protective behavior that evolved from long-term adaptation of myocardial cells to the environment, ensuring that the SIRT1-SIRT3 axis can provide continuous self-protection for myocardial cells to resist stress.

Previous studies have demonstrated that mitochondrial mass impairment and subsequent ferroptosis are key factors in MIRI [33]. Therefore, many studies have been conducted in various fields on the relationship between SIRT1/SIRT3 and ferroptosis [34, 35]. However, these studies mostly focused on either SIRT1 or SIRT3 individually. In addition, previous studies have presented two different perspectives on the relationship between SIRT1/SIRT3 and ferroptosis. Lee [36] believed that silencing histone deacetylase SIRT1 gene or pharmacological inhibition by EX-527 decreased ferroptosis, whereas the SIRT inducers Res and SRT1720 increased ferroptosis. Han [37] found that an increase in SIRT3 expression contributed to classic ferroptotic events and autophagy activation, whereas SIRT3 silencing led to resistance to ferroptosis and autophagy. However, contrary to the views of these two scholars, most scholars believe that the activation of SIRT1 and SIRT3 mitigates ferroptosis [34, 38–40]. The fact that these perspectives contradict each other indicates that although many studies have been performed on the relationship between SIRT1/SIRT3 and ferroptosis, there are still many unclear aspects. The present study confirmed that SIRT1 and SIRT3 formed an intrinsically related regulatory axis, which means that they are an integral whole. Therefore, we believe that it would prudent to conduct research on the relationship between SIRT1/SIRT3 and ferroptosis based on the SIRT1-SIRT3 axis as a whole. Therefore, the specific effects of the SIRT1-SIRT3 axis on myocardial cell ferroptosis were investigated during MIRI, and we found that the SIRT1-SIRT3 axis was closely related to the core DEGs (USP11, NQO1, and GSK3B) related to ferroptosis.

Subsequently, cell phenotype experiments showed that the SIRT1-SIRT3 axis silencing significantly increased the incidence of ferroptosis in cardiomyocytes. Carnevale et al. [12] believed that silencing SIRT1 increased not only the expression of SIRT3 but also the resistance of myocardial cells to stress, which means that SIRT1 does not have cardioprotective effect but rather affects their emergency resistance. However, our study showed that although silencing SIRT1 increased the expression of SIRT3, the ability of myocardial cells to resist stress was weakened. In addition, we showed for the first time that silencing SIRT3 was accompanied by an increase in the expression of SIRT1, but the stress resistance of myocardial cells was also weakened. As a result, knocking down SIRT1 or SIRT3 leads to a significant increase in the incidence of ferroptosis in myocardial cells, which means that although SIRT1 and SIRT3 mutually inhibit each other, either of them is indispensable in protecting cardiomyocytes against MIRI. This phenomenon further confirmed that the SIRT1-SIRT3 axis had clear overall properties, and any disruption in this axis will weaken the stress resistance of myocardial cells.

Ferroptosis is an autophagy-dependent and nonapoptotic form of cell death characterized by lipid peroxidation and Fe^2+^ accumulation [41]. In recent years, ferroptosis has received extensive attention because it is involved in the pathophysiological processes of tumor formation, kidney-related diseases, neurodegenerative diseases, stroke and other diseases [42]. The occurrence and development of ferroptosis are closely related to the pathological process of cardiomyocytes, and ferroptosis is involved in the pathogenic mechanisms of MIRI [43]. MIRI is characterized by Fe^2+^ deposition and ROS production, which can induce ferroptosis [27]. Studies have shown that USP22 can prevent MIRI through SIRT1-p53/SLC7A11-dependent inhibition of ferroptosis-induced cardiomyocyte death [20]. Intestinal SIRT1 deficiency protects mice from ethanol-induced liver injury by mitigating ferroptosis [44]. Resveratrol inhibits ferroptosis and decelerates heart failure progression via Sirt1/p53 pathway activation [45]. SIRT1 was one of the core genes related to ferroptosis in the progress of NASH [46]. NAC maintains mitochondrial redox homeostasis by activating SIRT3-SOD2-Gpx4 signaling pathway, thereby reducing ferroptosis in diabetic nephropathy [34]. Jing-Fang n-butanol extract and its isolated JFNE-C inhibit ferroptosis and inflammation in LPS induced RAW264.7 macrophages via STAT3/p53/SLC7A11 signaling pathway [47]. COVID-19 causes ferroptosis and oxidative stress in human endothelial cells [48]. Small extracellular vesicles from HO-1-modified bone marrow-derived mesenchymal stem cells attenuate ischemia-reperfusion injury after steatotic liver transplantation by suppressing ferroptosis via miR-214-3p [49]. Based on the findings of this study and previous studies, it is evident that the cardioprotective effect of the SIRT1-SIRT3 axis cannot be separated from its modulation of ferroptosis.

How does the SIRT1-SIRT3 axis modulate ferroptosis? Previous evidence has shown that changes in the mitophagy balance can affect the incidence of ferroptosis [50]. SIRT1 and SIRT3 are closely related to mitophagy. Inhibition of CDK9 can block the initiation of PINK1-PRKN-mediated mitochondrial phagocytosis by regulating the SIRT1-FOXO3-BNIP3 axis and enhance the therapeutic effect of mitochondrial dysfunction in hepatocellular carcinoma [51]. Activation of the SIRT1/PGC-1 pathway triggers mitophagy, which can alleviate oxidative damage in intestinal epithelial cells [52]. Melatonin improves excessive PINK1/Parkin-mediated mitophagy by enhancing the expression of SIRT1 [53]. Dexmetomidine alleviates hippocampal damage and cognitive impairment caused by liver ischemia/reperfusion in young rats by activating SIRT3-mediated mitophagy and inhibiting the activation of NLRP3 inflammasomes [54]. Omentin 1 improves myocardial ischemia-induced heart failure by relying on SIRT3/FOXO3a for mitochondrial dynamic homeostasis and mitophagy [55]. Therefore, we speculated that the SIRT1-SIRT3 axis can modulate ferroptosis by regulating the dynamic balance of mitophagy. However, the regulatory mechanism of mitophagy balance is complex and involves numerous regulatory pathways. At present, there are two main mechanisms that regulate mitophagy: the Parkin-dependent pathway, which is mediated by Parkin and the mitochondrial outer membrane protein PINK1 [56], and the Parkin-independent pathway, which includes FUNDC1-mediated mitophagy and Binp3/Nix-mediated mitophagy [57]. SIRT1/SIRT3 participate in many autophagy regulatory pathways, such as AMPK, PINK1/Parkin, SIRT1/PGC-1, and SIRT3/FOXO3a [52, 53, 55, 58]. As yet, there is no consensus on which pathway the SIRT1-SIRT3 axis participates in to regulate mitophagy. In this study, bioinformatics analysis showed that the SIRT1-SIRT3 axis was closely correlated with PINK1, Parkin, P62/SQSTM1 and LC3, which means that the PINK1/Parkin signaling pathway may be important for the regulation of the SIRT1-SIRT3 axis. Recently, studies indicated that PINK1 and Parkin are important components in the regulation of mitophagy and the maintenance of normal mitochondrial function [59].PINK1 is a protein kinase, that is mainly located in the inner membrane of mitochondria, while Parkin is an E3 ubiquitin ligase, that is mainly located in the cytoplasm [60]. A Drosophila knockout model experiment first clarified the interaction between PINK1 and Parkin [61]. These factors jointly regulate mitophagy to maintain mitochondrial quality. It has been confirmed that PINK1 can be transferred to mitochondria through Parkin phosphorylation [62]. Parkin ubiquitinates outer membrane proteins of mitochondria, which further triggers mitophagy and clears all labeled cells by autophagy [62]. The formation of isolation membranes around damaged mitochondria is mediated by autophagic receptors, such as P62/SQSTM1 [62–64]. When autophagy occurs, P62/SQSTM1 is preferentially localized to adjacent damaged mitochondria and promotes the aggregation of damaged mitochondria through its PBI oligomeric domain [63]. Finally, the formation of autophagic lysosomes is completed with the help of LC3 [65]. LC3 is a microtubule-associated protein, whose carboxyl end is immediately cleaved by Agt4 after synthesis to form cytoplasmic LC3-I with a molecular size of 16 kDa [66]. After autophagy occurs, LC3-I is further modified and processed by Agt3 and Agt7 to form LC3-II with a molecular weight of 14 kDa, which is localized to autophagosomes to assist in autophagy [66]. Therefore, the LC3-II/I ratio can be used to evaluate the level of autophagy. Based on the functions of these genes and their role in autophagy, changes in their expression reflect changes in the dynamic balance of mitophagy [23, 24].

To further demonstrate that the SIRT1-SIRT3 axis modulates ferroptosis via the PINK1/Parkin signaling pathway, we silenced PINK1, the key molecule of this pathway. After gene silencing, we were surprised to find that the same ferroptotic phenotypes as the SIRT1-SIRT3 axis silencing were observed. Moreover, Res and HKL could effectively reverse the effects of PINK1 gene silencing, especially HKL. Res and HKL, which are well-established specific activators of SIRT1 and SIRT3, can increase the expression of SIRT1 and SIRT3, respectively [67–69], which suggests that upregulating the SIRT1-SIRT3 axis can promote the expression of PINK1 to inhibit ferroptosis caused by PINK1 silencing. Therefore, the results confirmed the relationship among the SIRT1-SIRT3 axis, ferroptosis and the PINK1/Parkin signaling pathway.

In summary, our findings improve out understanding of the cardioprotective effect mediated by the SIRT1-SIRT3 axis and clarify the underlying mechanisms by which abnormalities in this axis cause MIRI. The results suggest that the SIRT1-SIRT3 axis might offer some promising prospects for the effective prevention and treatment of MIRI in patients with ICM. Despite these promising results, there are several limitations to this study. First, this study was carried out in combination with bioinformatics analysis and cell experiments, and the results were not verified by animal models. Second, although we observed that SIRT1 and SIRT3 exhibited intrinsic mutual inhibition, further research is needed in the future to understand why they formed such interactions.

## Conclusions

The data in the present study demonstrated that the expression level of SIRT1 and SIRT3 was abnormal when cardiomyocytes suffered from MIRI. Moreover, we confirmed for the first time that although SIRT1 and SIRT3 inhibit each other, they are both indispensable. The SIRT1-SIRT3 axis has obvious overall properties and any disruption in this axis would weaken the stress resistance of myocardial cells. Abnormalities in the SIRT1-SIRT3 axis promote MIRI through ferroptosis caused by silencing the PINK1/Parkin signaling pathway. Therefore, the present study provides some new findings and insights into the roles of SIRT1 and SIRT3 in MIRI, which may provide a theoretical basis and potential targets for the effective prevention and treatment of MIRI in patients with ICM.

### Electronic supplementary material

Below is the link to the electronic supplementary material.

Supplementary Material 1

Supplementary Material 2

Supplementary Material 3

Supplementary Material 4

Supplementary Material 5

## Data Availability

The datasets used and/or analyzed during the current study are available from the corresponding author upon reasonable request.
