# Supplementary material for: Abnormalities in the SIRT1-SIRT3 axis promote myocardial ischemia-reperfusion injury through ferroptosis caused by silencing the PINK1/Parkin signaling pathway
Source: BMC Cardiovasc Disord. 2023 Nov 27;23:582. doi: 10.1186/s12872-023-03603-2 (PMC10683361; doi:10.1186/s12872-023-03603-2)
Supplement: Supplementary file 4 — Supplementary Material 4 [file 12872_2023_3603_MOESM4_ESM.doc]

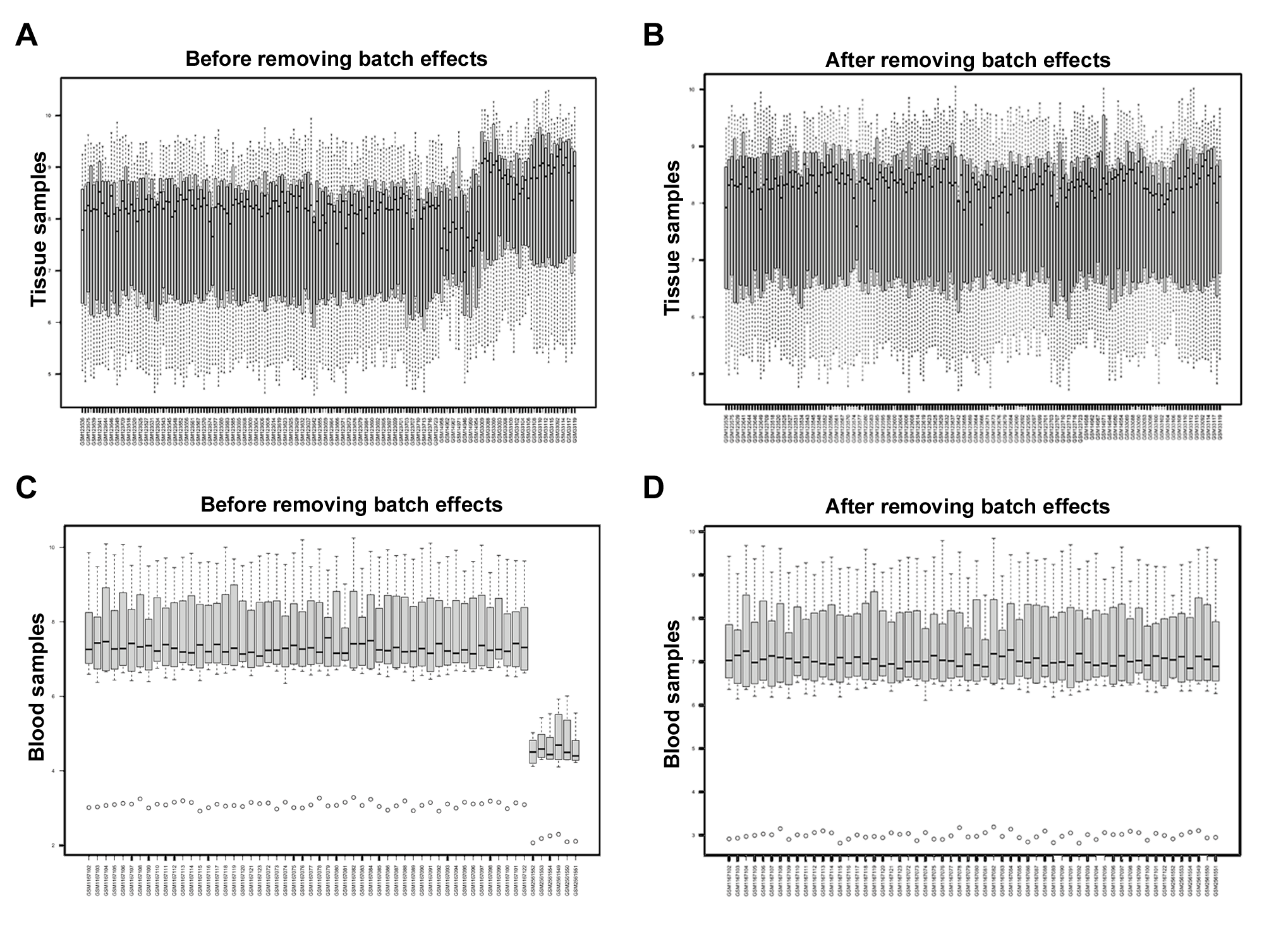


**Figure S1** Comparison of data homogeneity before and after the batch effects were removed. (**A, B)** Data homogeneity comparison of merged datasets derived from tissue samples before and after the batch effects were removed. (**C, D)** Data homogeneity comparison of merged datasets derived from peripheral blood samples before and after the batch effects were removed.
