# Supplementary material for: Abnormalities in the SIRT1-SIRT3 axis promote myocardial ischemia-reperfusion injury through ferroptosis caused by silencing the PINK1/Parkin signaling pathway
Source: BMC Cardiovasc Disord. 2023 Nov 27;23:582. doi: 10.1186/s12872-023-03603-2 (PMC10683361; doi:10.1186/s12872-023-03603-2)

Original images of Figure 2B

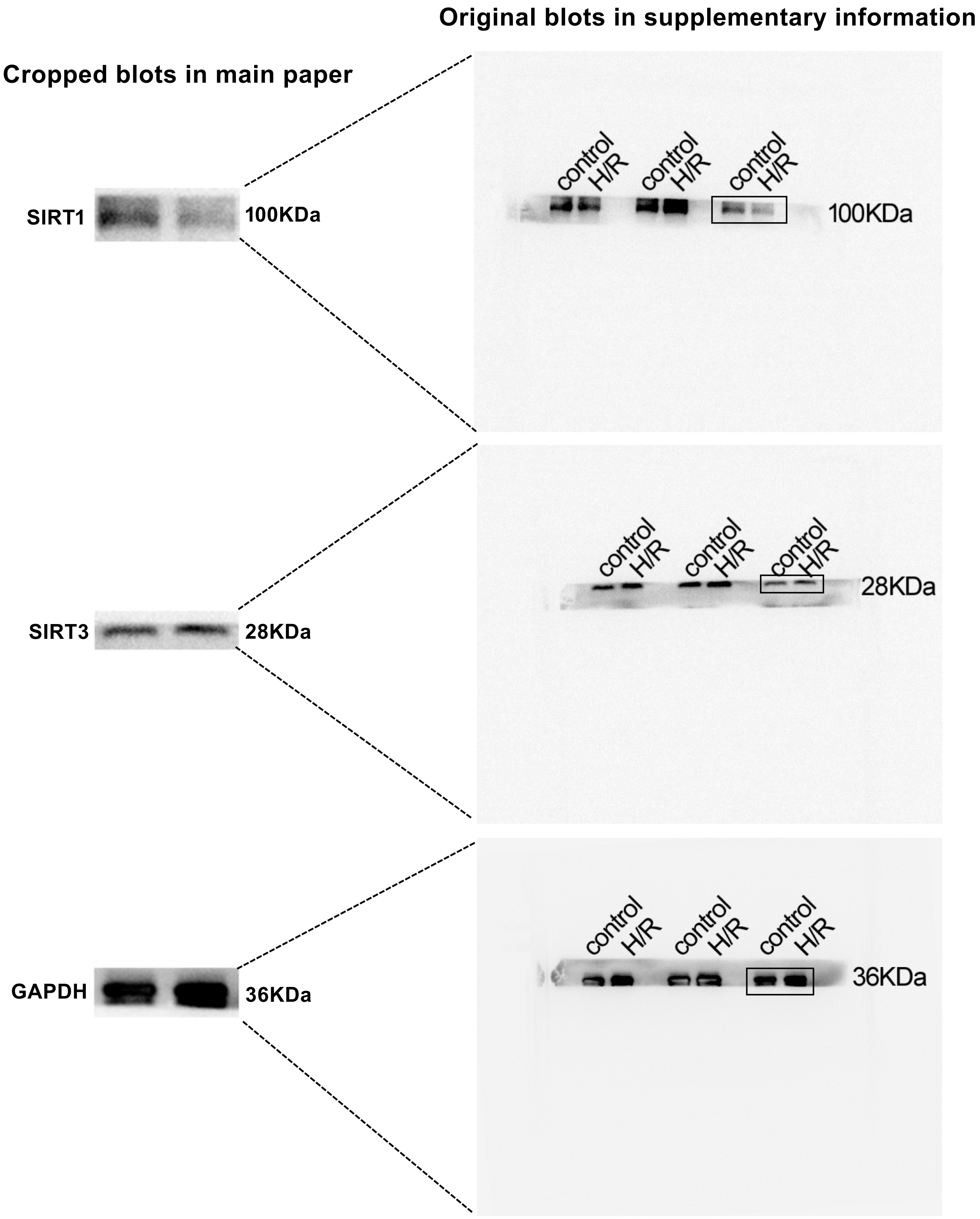

Original images of Figure 2D

Original blots in supplementary information

Cropped blots in main paper

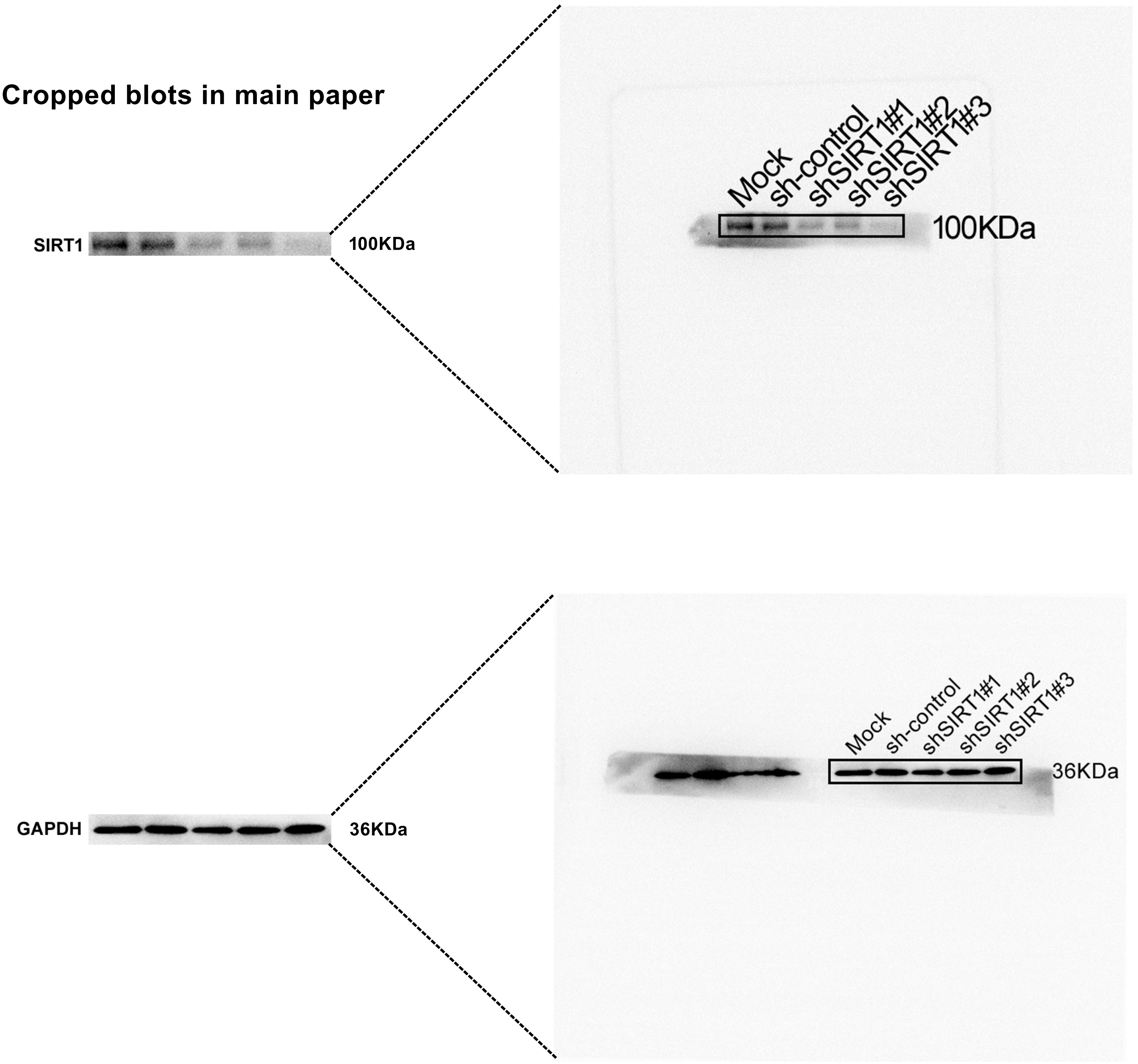

Original images of Figure 2E

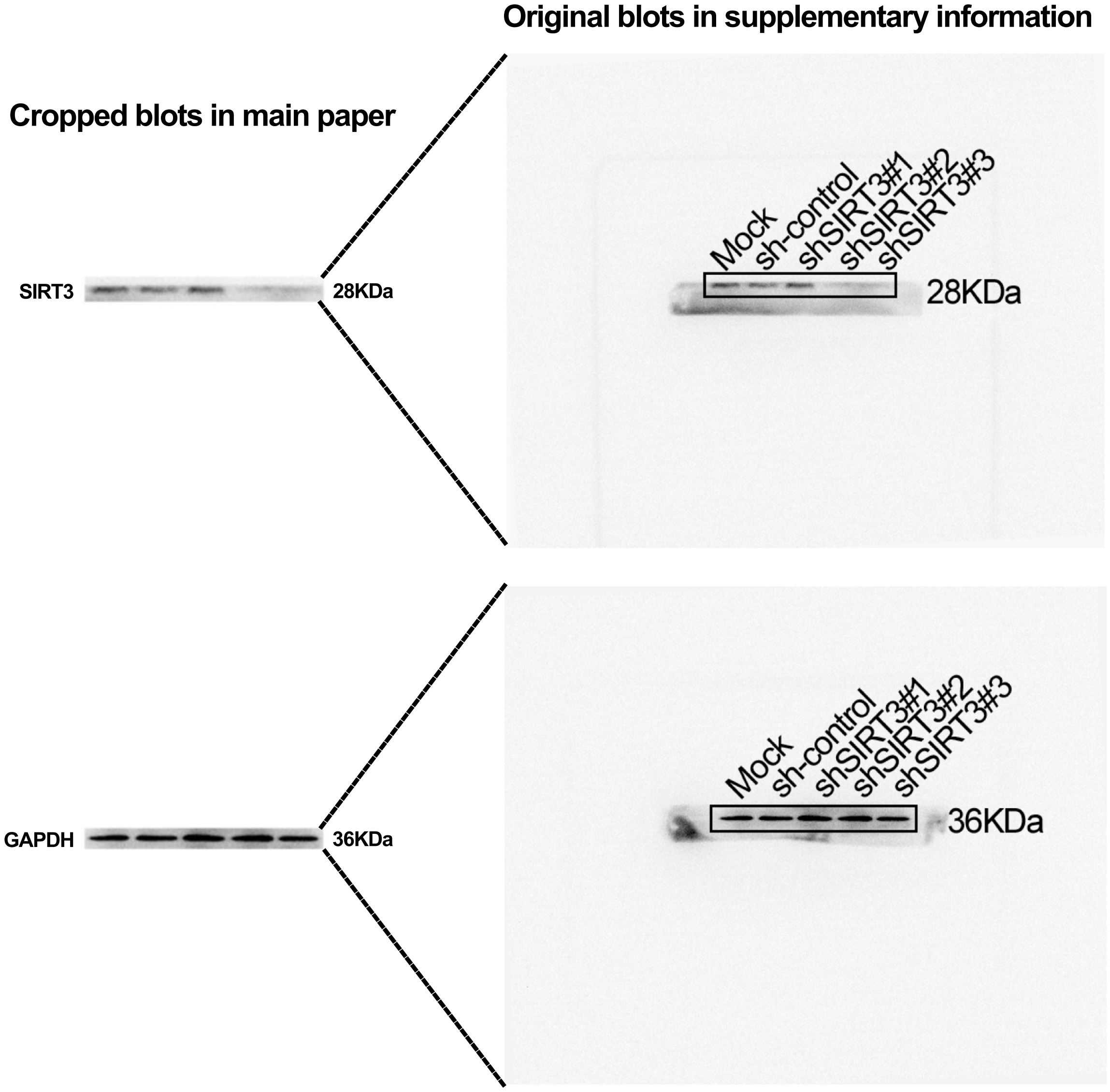

Original images of Figure 2F

Original blots in supplementary information

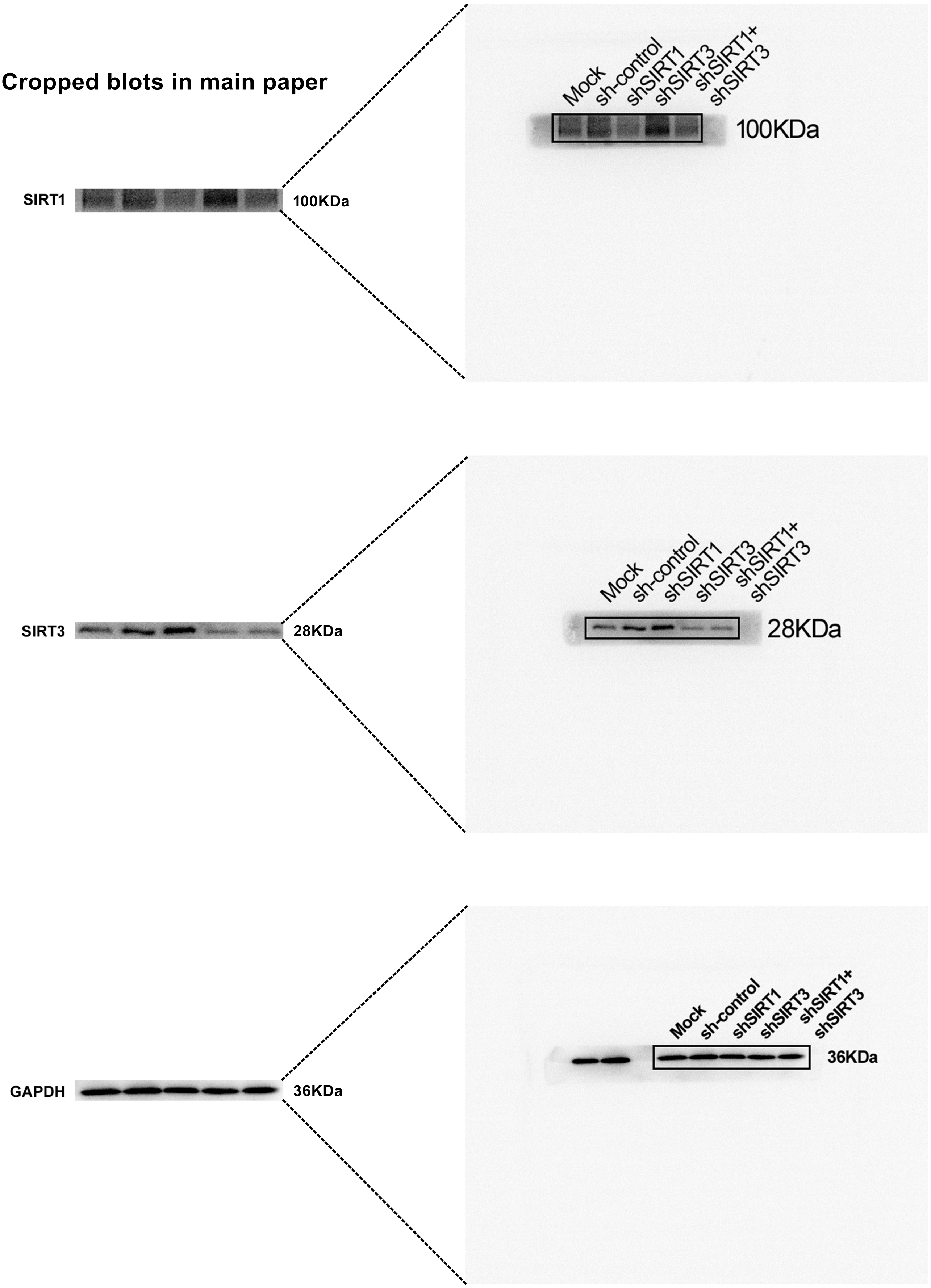

Original images of Figure 5B

Original blots in supplementary information

Cropped blots in main paper

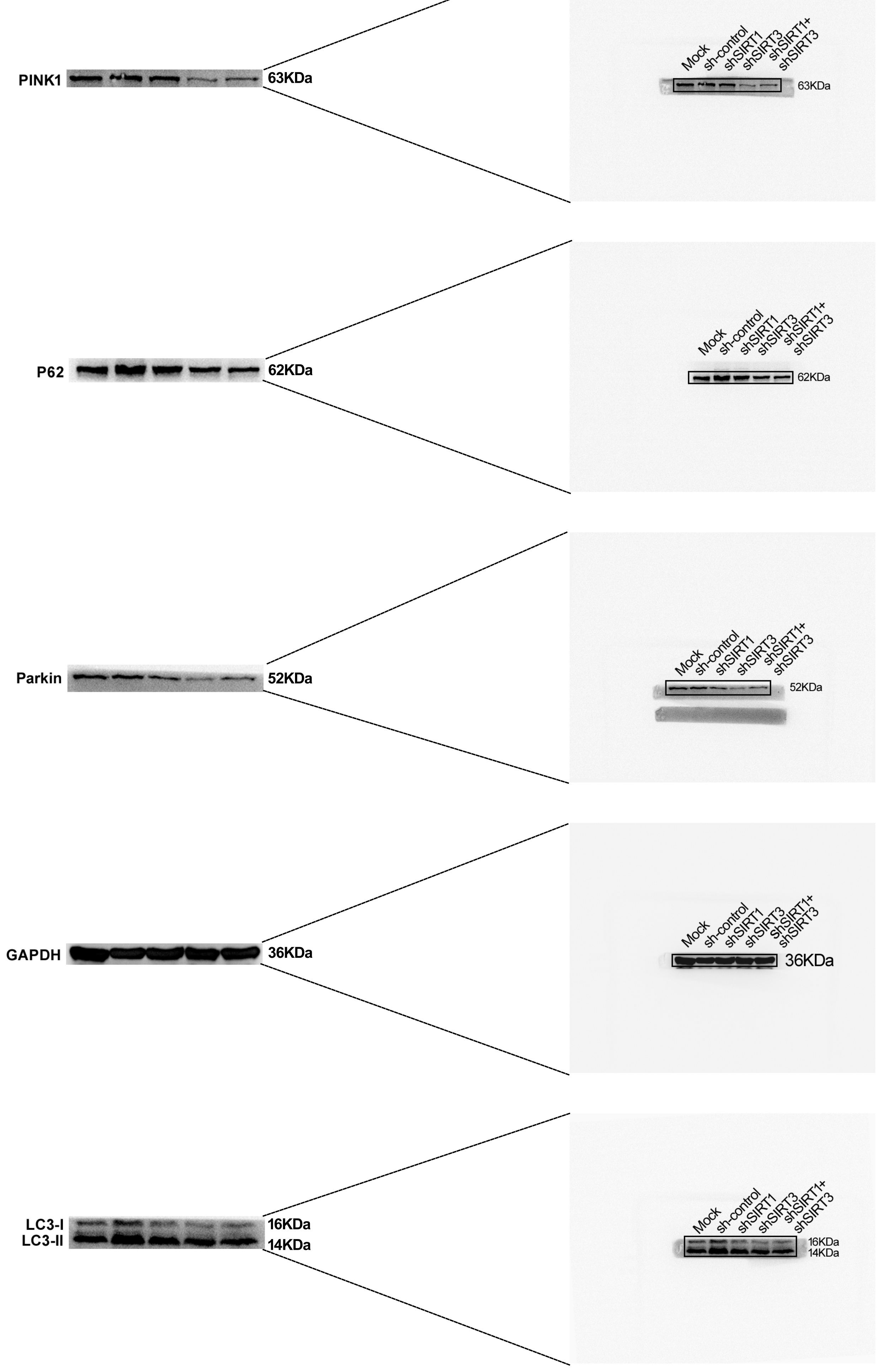

Original images of Figure 6A

Original blots in supplementary information

Cropped blots in main paper

PINK1 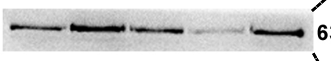 63kDa

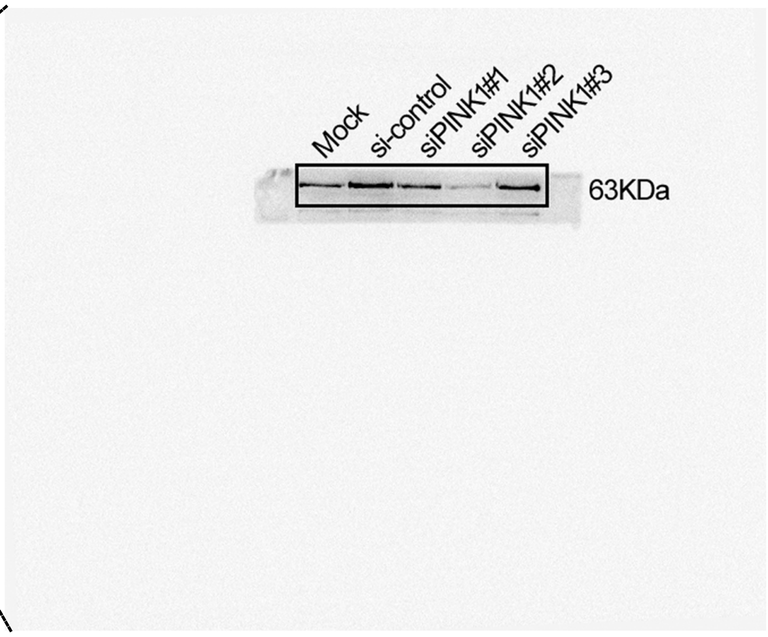

GAPDH 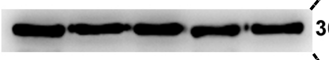 36kDa

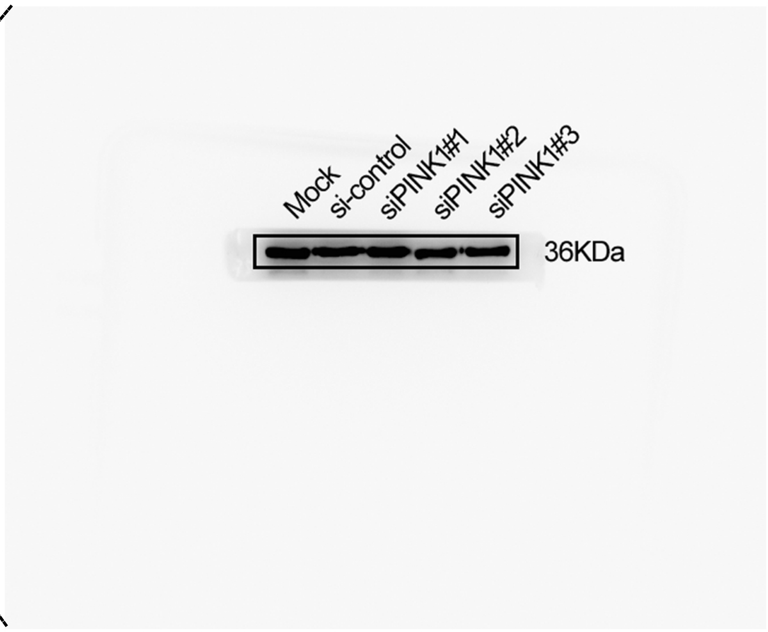

Original images of Figure 6C

Original blots in supplementary information

Cropped blots in main paper

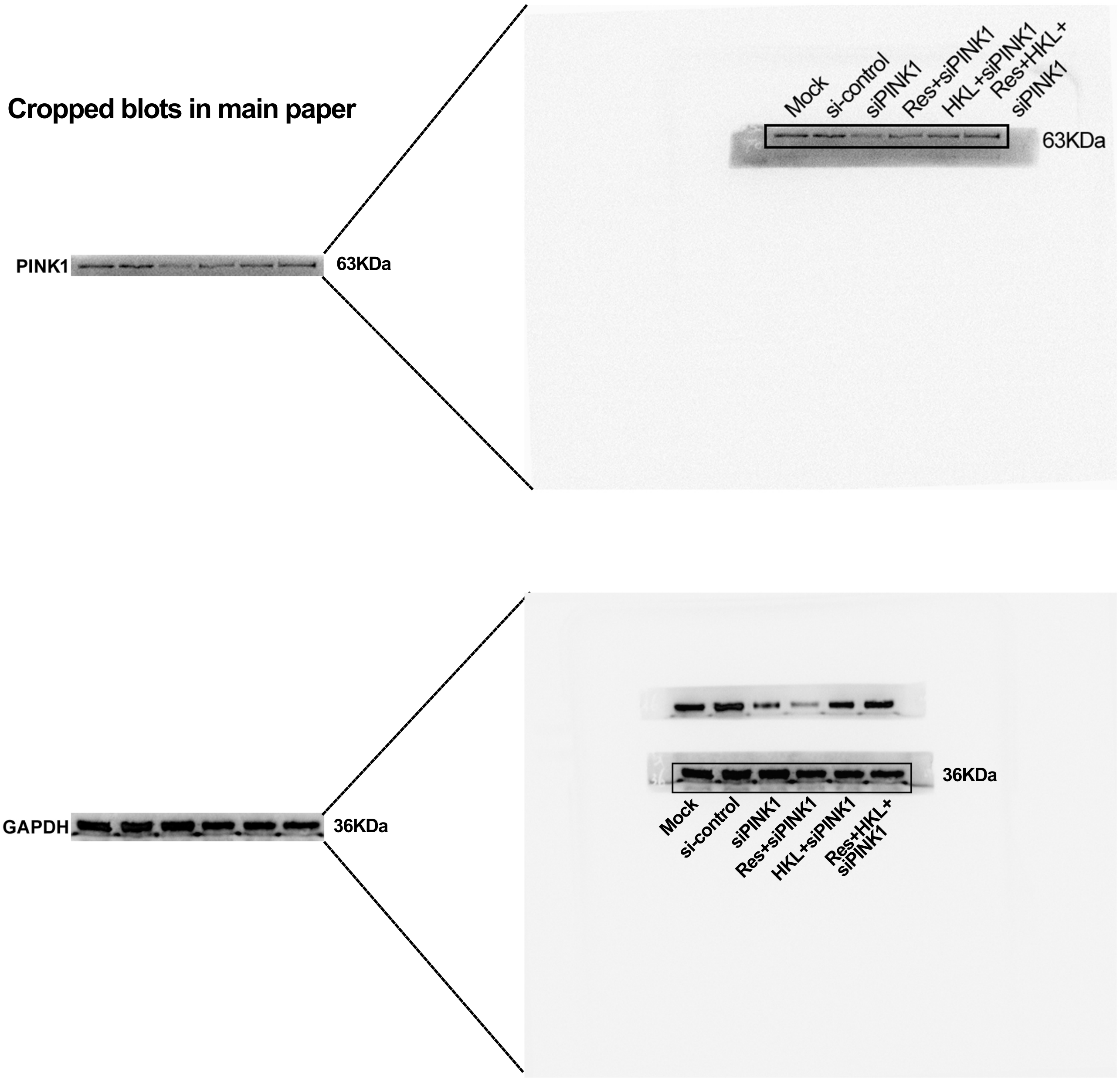

Supplement: Supplementary file 5 — Supplementary Material 5 [file 12872_2023_3603_MOESM5_ESM.pdf]
